# Assessing stroke recurrence in sICAS: a study on mCSVD score and culprit plaque magnetic resonance characteristics

**DOI:** 10.3389/fneur.2024.1478583

**Published:** 2024-11-19

**Authors:** Kaixuan Ren, Juan He, Li Zhu, Yue Gu, Hang Qu, Yi Zhao, Wei Wang

**Affiliations:** ^1^Department of Medical Imaging, The Affiliated Hospital of Yangzhou University, Yangzhou University, Yangzhou, China; ^2^Department of Neurology, The Affiliated Hospital of Yangzhou University, Yangzhou University, Yangzhou, China; ^3^Department of Medical Imaging, The Second Affiliated Hospital of Nantong University, Nantong, China

**Keywords:** small vessel disease burden, stroke recurrence, vessel wall imaging MRI, acute ischemic stroke, intracranial atherosclerotic stenosis

## Abstract

**Background:**

Recurrent ischemic stroke in patients with symptomatic intracranial atherosclerotic stenosis (sICAS) can be attributed to two main causes: intracranial atherosclerotic stenosis (ICAS) and cerebral small vessel disease (CSVD). This study investigates the potential associations between stroke recurrence and the modified cerebral small vessel disease (mCSVD) burden score, as well as the characteristics of culprit plaques related to intracranial artery high-resolution vessel wall imaging (HR-VWI).

**Methods:**

A total of 145 patients presenting sICAS underwent intracranial artery HR-VWI and routine cranial MRI at two large Chinese hospitals from December 2019–2022 were participants of this retrospective analysis. Standard MRI scans were used to calculate the mCSVD score. Following a 12-month observation period, the patients were categorized into two distinct groups depending on whether or not they experienced a subsequent stroke.

**Results:**

Within 12 months, 32 patients experienced stroke recurrence. The recurrence group’s mCSVD score was higher compared to the non-recurrence group (*p* < 0.001). Their luminal stenosis and culprit plaque thickness and burden were also higher (*p* < 0.05). Additionally, higher rates of diabetes, T1WI hyperintensity of culprit plaques, and significant plaque enhancement were observed in the recurrence group (*p* < 0.05). The adjusted Cox regression model indicated that the mCSVD score (HR = 1.730, 95% CI 1.021–2.933, *p* = 0.042) and T1WI hyperintensity of the culprit plaque (HR = 6.568, 95% CI 1.104–39.059, *p* = 0.039) remained significantly independent risk variables. The combination of the mCSVD score and T1WI hyperintensity of the culprit plaque demonstrated the highest efficacy in predicting stroke recurrence (*z* = 2.678, *p* < 0.05).

**Conclusion:**

The mCSVD score, associated with T1WI hyperintensity of culprit plaque, effectively predicts stroke recurrence and can be easily obtained, offering high clinical value.

## Introduction

Intracranial atherosclerotic stenosis (ICAS) is a major causative factor for ischemic stroke worldwide. Patients with symptomatic ICAS experience stroke recurrence risk between 20 and 30%. The rates of morbidity and mortality associated with recurrent strokes are significantly higher than those observed after the initial onset (1, 2). Research has shown a strong association between the features of intracranial arterial culprit plaques, as detected by high-resolution vessel wall imaging (HR-VWI), and the recurrence of stroke (3–5). Many studies have primarily examined the characteristics of intracranial large arteries, overlooking the effect of cerebral small vessel disease (CSVD) burden on stroke recurrence.

CSVD involves brain damage from cerebral arterioles, veins, and capillaries, leading to clinical, imaging, and pathological changes (6, 7). Multiple CSVD imaging biomarkers are frequently detected in individuals with stroke, and CSVD heightens the possibility of stroke occurrence and recurrence (8). Currently, the presence of lacunes, cerebral microbleeds (CMBs), enlarged perivascular spaces (EPVS), and white matter hyperintensity (WMH) was used to compute the total CSVD burden score (tCSVD score; 0–4 points) (9). It has been demonstrated that the tCSVD score is a predictive measure of cognitive impairment and stroke recurrence (10, 11). Nevertheless, this scoring method is relatively simple and does not adequately capture the severity of each imaging biomarker. Amin et al. developed a modified CSVD burden score (mCSVD score) with a maximum of 7 points, incorporating both the overall burden of CSVD and the severity of individual imaging biomarkers. Their research, which was confirmed by three cohort studies, showed that the modified cerebral small vessel disease (mCSVD) score improved the ability to predict dementia and cognitive decline compared to the traditional CSVD (tCSVD) score (12). However, to evaluate the predictive value of this score for stroke recurrence, more research is necessary.

This study hypothesizes that integrating intracranial arterial plaque characteristics with the mCSVD score may improve the prediction of stroke recurrence in patients. This research uses HR-VWI to evaluate plaque characteristics and the mCSVD score to assess the overall burden of CSVD in the brain. The study aims to develop a predictive model and evaluate its efficacy in predicting stroke recurrence in ischemic stroke patients, thereby equipping clinicians with a tool for early assessment of recurrence risk.

## Methods

### Patient data, inclusion, and exclusion criteria

**Figure 1:**
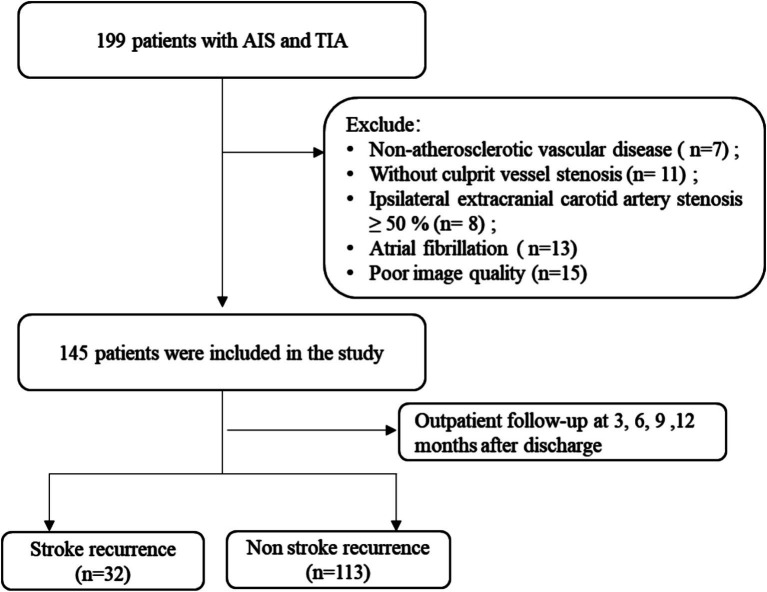
Schematic representation of the process for enrolling patients. AIS, acute ischemic stroke; TIA, transient ischemic attack.

The ischemic stroke patients (*n* = 199) were admitted to our hospital’s Department of Neurology between December 2019 and December 2022 and were the participants of a retrospective analysis. Magnetic resonance angiography (MRA), electrocardiogram (ECG), carotid ultrasound, and magnetic resonance imaging (MRI) were among the diagnostic assessments that each patient received. Within 1 week, an HR-VWI examination was conducted in cases where these diagnostic techniques identified the stroke under the TOAST criteria as large artery atherosclerosis or suggested that intracranial atherosclerosis was the cause of the ischemic event. The availability of all baseline data and the finding of the HR-VWI assessment indicating plaque development in all culprit’s vessels were the inclusion criteria for this investigation. All patients received conservative therapy with basic pharmacological interventions during hospitalization. The following were the exclusion criteria: (1) non-atherosclerotic vascular conditions, such as vasculitis of the primary nervous system (5 cases) or vascular malformation (2 cases); (2) cases of the culprit vessels where stenosis was absent (11 cases); (3) 50% or more of the ipsilateral extracranial carotid artery stenosis (8 cases); (4) possibility of cardiogenic embolism risk factors, including atrial fibrillation (13 cases); and (5) low image quality (15 cases). The unit’s ethical committee authorized the trial, which involved 145 patients after the consent of the participants or their legal guardians was obtained (Figure 1).

### Data collection

Gender, age, smoking, diabetes, hypertension, and coronary heart disorder were among the baseline data that were collected. Moreover, a venous blood sample (5 mL) of individuals who had fasted was acquired on the second day of post-hospitalization to evaluate blood lipids, high-sensitivity C-reactive protein, lipoprotein-associated phospholipase A2, fasting blood glucose, homocysteine, and cystatin C.

### mCSVD score assessment

Following the international imaging standard for cerebral small vessel disease 2 (STRIVE-2) (6) and the results of MRI examinations conducted during hospitalization, the mCSVD score was assessed. According to the number of lacunes, they were given a score between 0 and 3 (0 = none, 1 = 1–2, 2 = 3–5, and 3 = more than 5). White matter hyperintensities (WMH) were scored from 0 to 3 according to the Fazekas scale. If cerebral microbleeds (CMBs) were found, they received a score of 1. The cumulative mCSVD burden score totaled 7 points.

### HR-VWI protocol

The Siemens Verio 3.0T MR imaging equipment (Siemens, Erlangen, Germany) was utilized in this work. The scanning protocol included magnetic resonance time-of-flight angiography (TOF-MRA), diffusion-weighted imaging (DWI), T2 fluid-attenuated inversion recovery (T2-FLAIR), susceptibility-weighted imaging (SWI), and three-dimensional T1 variable flip angle fast spin echo (3D-T1-SPACE). 3D-T1-SPACE sequence performed both pre-and post-injection of the gadoxetic acid (contrast agent). The scan lasted for 7 min and 16 s. The contrast agent was administered with 0.1 mmol/kg. The whole presentation of the bilateral internal carotid artery, the anterior cerebral artery, the middle cerebral artery, the posterior cerebral artery, the basilar artery, the intracranial segment of the vertebral artery, and the P1–P2 segment of the posterior cerebral artery were all included in the scanning range.

### Culprit plaque characteristics assessment

**Figure 2:**
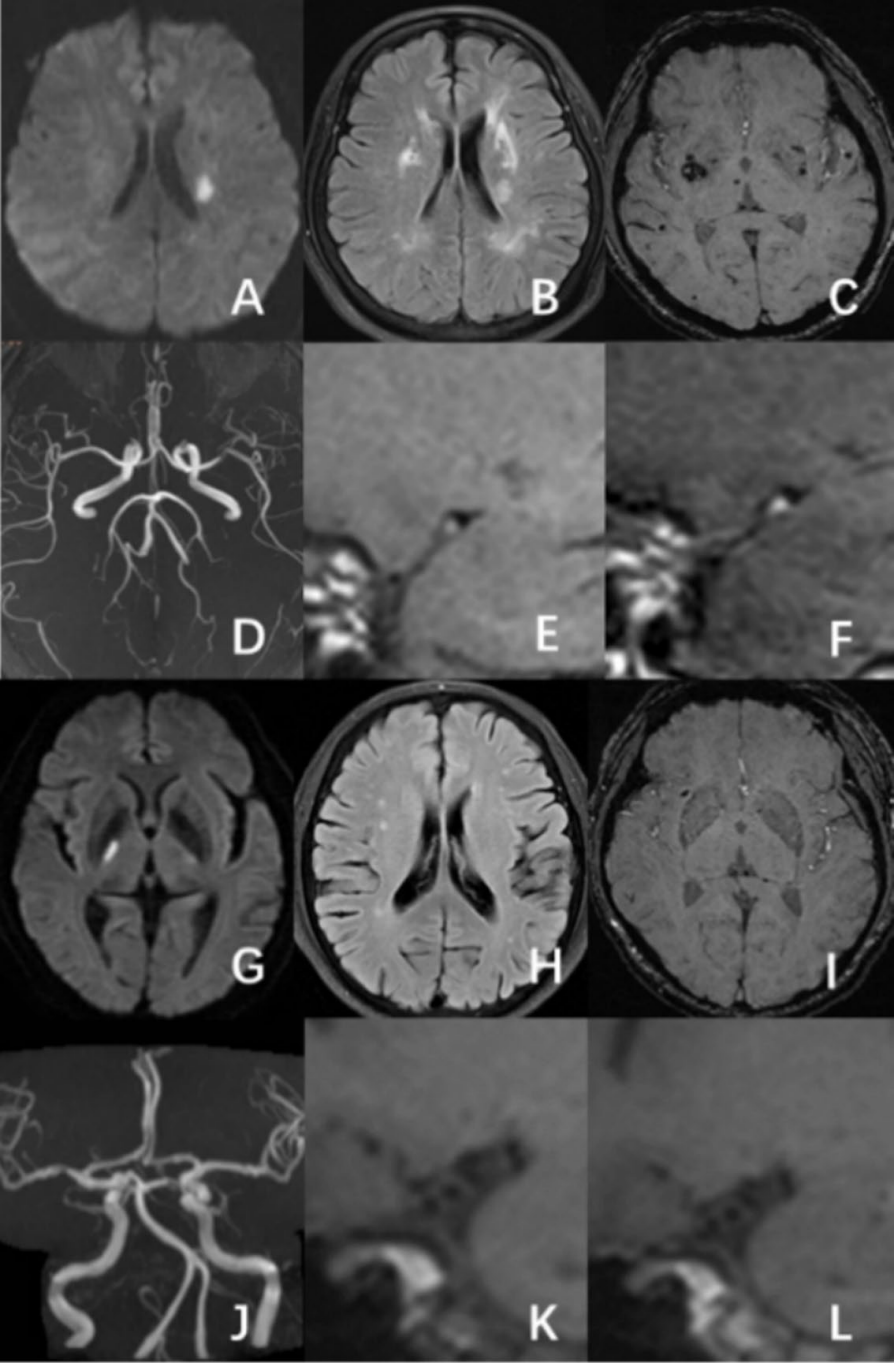
A male patient, aged 58, had an acute ischemic stroke in the left basal ganglia area, with a mCSVD score of 5 points. HR-VWI demonstrated hyperintensity on T1WI and significant enhancement on the culprit plaque present at the left middle cerebral artery **(A–F)**. A 65-year-old male patient was experiencing an acute ischemic stroke in the right thalamus. The patient has a mCSVD score of 2 points. HR-VWI revealed T1WI iso-intensity and mild enhancement in the right middle cerebral artery culprit plaque **(G–L)**.

Based on DWI symptoms and signs of acute infarction or clinical neurological impairments that were reported, the culprit vessels were identified. The culprit plaque was determined to produce the largest stenosis of the culprit vessel on HR-VWI. Culprit plaque analysis involved measuring plaque thickness, residual lumen area, plaque burden, and lumen stenosis, evaluating the T1WI hyperintensity, positive remodeling, and significant plaque enhancement (13). A five-year-experienced neuroradiologist and a second-year graduate student in neuroimaging evaluated all images. By consulting with a third neuroradiologist with 10 years of expertise, disagreements were settled (Figure 2).

### Stroke recurrence assessment

Upon discharge, all patients were prescribed antiplatelet and lipid-lowering drugs for an average duration of 25 days or more each month. Outpatient follow-up assessments were carried out three, six, nine, and twelve months after discharge. DWI showed the existence of additional acute infarcts in the same vascular area, which was used to diagnose stroke recurrence. In the absence of imaging data for suspected stroke recurrence, follow-up clinicians determined the occurrence of outcome events depending on the presence of new neurological impairment symptoms characterized by sudden neurological deterioration. It was considered neurological deterioration when new neurological problems continued for more than 24 h or when the National Institutes of Health Stroke Scale (NIHSS) rose by more than 4 points. The time interval between discharge and the endpoint event’s occurrence was designated as the recurrence time. In cases where no endpoint event occurred, it was defined as the duration from discharge to the last outpatient follow-up (14).

### Statistical analysis

The SPSS software (version 21.0; IBM Corp., Armonk, NY, USA) was used in the present study for all statistical analysis. With the Kolmogorov–Smirnov test, the distribution of the data was assessed for normality. Variables were analyzed and compared using the *t*-test, with a normal distribution, and indicated as mean ± standard deviation (SD). When a variable did not have a normal distribution, it was presented as the median (interquartile range), and the Mann–Whitney U test was used for comparison. The data of the counts were analyzed via chi-square analysis and displayed as frequency (percentage). The mCSVD burden score was compared between the groups with and without recurrence using the rank sum test. The variables with a *p*-value less than 0.05 were incorporated from the univariate analysis into the multivariate Cox regression model, using the backward stepwise approach to ascertain independent variables. Variables including age, gender, hypertension history, smoking history, and those found in the univariate analysis with a *p*-value less than 0.05 were included in the binary logistic regression analysis to calculate propensity scores. The propensity score covariate adjustment approach was used to mitigate confounding variables in the prediction model. The model’s performance was evaluated via the Receiver Operating Characteristic (ROC) curve and the area under the curve (AUC). A *p*-value of less than 0.05 was considered statistically significant.

## Results

A total of 145 patients (96 = males; 49 = females) with symptomatic intracranial atherosclerotic stenosis were included in this study. The mean age of the participants was 63 ± 12 years. Notably, 22.1% (32 out of 145) of the patients experienced stroke recurrence within 12 months. Diabetes was much more common in the recurrent group in relation to the group without recurrence (53.1% vs. 27.1%, *p* = 0.010). Additionally, the levels of fasting blood glucose and glycosylated hemoglobin comparing the two groups differed significantly (*p* < 0.05). As shown in Table 1, the recurrence group’s serum HDL levels were significantly (*p* < 0.05) lower in relation to the group without recurrence.

**Table 1:** The baseline clinical data of the non-recurrence and recurrence groups of the study participants.

Characteristic	Non-recurrence group (*n* = 113)	Recurrence group (*n* = 32)	*t/z/χ^2^*	*p-*value
Age [y]	62.82 ± 12.93	65.58 ± 9.84	−1.102	0.272
Male [n (%)]	71 (62.8)	25 (78.1)	−2.607	0.139
Smoking history [n (%)]	25 (22.1)	12 (37.5)	−3.102	0.107
History of hypertension [n (%)]	84 (74.3)	19 (51.4)	−2.713	0.123
History of diabetes [n (%)]	31 (27.4)	17 (53.1)	−7.433	0.010
Admission NIHSS score	2.86 ± 3.77	3.12 ± 3.49	−0.315	0.753
FBG (mmol/L)	6.08 ± 2.55	7.26 ± 2.50	−2.269	0.025
HbA1c (mmol/L)	6.73 ± 1.33	7.32 ± 1.67	−2.012	0.046
TG (mmol/L)	1.68 ± 1.11	1.75 ± 1.00	−0.282	0.778
TC (mmol/L)	4.14 ± 1.05	3.98 ± 1.37	0.681	0.562
HDL (mmol/L)	1.21 ± 0.34	1.07 ± 0.26	2.112	0.037
LDL (mmol/L)	3.33 ± 7.46	2.51 ± 1.26	0.595	0.553
Apolipoprotein A/B	1.33 ± 0.46	1.38 ± 0.59	−0.443	0.658
Lp-PLa2 (ng/ml)	353.86 ± 217.37	327.89 ± 175.31	0.572	0.568
hs-CRP (mg/L)	3.26 ± 10.10	4.51 ± 14.91	−0.734	0.464
Hcy (μmol/L)	14.00 ± 8.08	12.32 ± 4.02	1.397	0.163
Cystatin-C (pg/mL)	21.01 ± 111.61	29.03 ± 139.02	−0.312	0.756

*p < 0.05 = statistically significant result.

FBG, fasting blood glucose; NIHSS, national institutes of health stroke scale; Lp-PLa2, lipoprotein-associated phospholipase a2; HbA1c, glycosylated hemoglobin; TC, total cholesterol; TG, triglyceride; LDL, low-density lipoprotein; HDL, high-density lipoprotein; Hcy, homocysteine; hs-CRP, high-sensitivity C-reactive protein.

The recurrence group exhibited a higher number of lacunes, a greater WMH Fazekas grade, and an increased count of CMBs than the non-recurrence group, showing significant outcomes (all *p* < 0.05). As shown in Table 2, a significant difference in the median mCSVD load score was present between both groups, with the recurrence group scoring 4 points and the non-recurrence scoring 2 (*p* < 0.001).

**Table 2:** Comparison of the CSVD imaging biomarkers and mCSVD score between the recurrence and non-recurrence groups of patients.

Characteristic score	Non-recurrence group (*n* = 113)	Recurrence group (*n* = 32)	*χ*″	*p-*value
Lacune [n (%)]			17.121	0.001
0	67 (59.3)	8 (25.0)		
1	26 (23.0)	9 (28.1)		
2	18 (15.9)	11 (34.4)		
3	2 (1.8)	4 (12.5)		
WMH [n (%)]			11.05	0.011
0	19 (16.8)	1 (3.1)		
1	54 (47.8)	10 (31.3)		
2	39 (34.5)	21 (65.6)		
3	1 (0.9)	0 (0)		
CMBs [n (%)]			10.884	0.001
0	69 (61.1)	44 (38.9)		
1	9 (28.1)	23 (71.9)		
mCSVD score	2 (1, 3)	4 (3, 5)	−4.843	< 0.001

*p < 0.05 = statistically significant result.

WMH, white matter hyperintensity; CMBs, cerebral microbleeds.

No significant disparity was observed in the proportion of anterior circulation between the two groups (50.0% vs. 57.5%, *p* = 0.546), nor did the proportion of AIS patients (81.3% vs. 67.3%, *p* = 0.187). The plaque thickness (1.75 ± 0.53 vs. 1.50 ± 0.36), plaque burden (77.53 ± 10.26 vs. 70.81 ± 10.12), and lumen stenosis degree (51.81 ± 21.29 vs. 39.62 ± 17.21) of the recurrence group were reported to be significantly greater in relation to the group without recurrence (all *p* < 0.05). In comparison to the non-recurrence group, the recurrence group had a higher incidence of T1WI hyperintensity (53.1% vs. 21.2%, *p* = 0.001). Table 3 shows that the recurrent group had a higher incidence of significant plaque enhancement (53.1% vs. 25.7%, *p* = 0.010) than the non-recurrent group.

**Table 3:** Comparison of the culprit plaques in the recurrence and non-recurrence groups.

Characteristic	Non-recurrence group (*n* = 113)	Recurrence group (*n* = 32)	*t/z/χ^2^*	*p-*value
AIS [n (%)]	76 (67.3)	26 (81.3)	2.341	0.187
Plaque thickness (mm)	1.50 ± 0.36	1.75 ± 0.53	−2.467	0.018
Remaining lumen area (mm^2^)	4.47 ± 2.58	4.43 ± 3.89	0.188	0.852
Degree of stenosis (%)	39.62 ± 17.21	51.81 ± 21.29	−3.350	0.001
Plaque burden (%)	70.81 ± 10.12	77.53 ± 10.26	−3.309	0.001
Significant enhancement [n (%)]	29 (25.7)	17 (53.1)	6.861	0.010
T1WI hyperintensity [n (%)]	24 (21.2)	17 (53.1)	12.502	0.001

*p < 0.05 = statistically significant result.

AIS, acute ischemic stroke; T1WI, T1 weight imaging.

After stepwise backward selection, three variables were eventually retained in the Cox regression model: HDL, mCSVD score, and T1WI hyperintensity of the culprit plaque. The mCSVD score (HR = 1.730, 95% CI 1.021 ~ 2.933, *p* = 0.042) and T1WI hyperintensity of the culprit plaque (HR = 6.568, 95% CI 1.104 ~ 39.059, *p* = 0.039) were significant independent risk factors after adjusting the Cox regression model for covariate propensity score (Table 4).

**Table 4:** Cox regression analysis of stroke recurrence risk variables.

Characteristic	Unadjusted		Adjusted	
	HR (95% CI)	*p* value	HR (95% CI)	*p* value
HDL	0.377 (0.105 ~ 1.356)	0.135	0.349 (0.065 ~ 1.884)	0.221
mCSVD score	1.675 (1.288 ~ 2.179)	<0.001	1.730 (1.021 ~ 2.933)	0.042
T1WI hyperintensity	5.868 (2,565 ~ 13.427)	<0.001	6.568 (1.104 ~ 39.059)	0.039

*p < 0.05 = statistically significant result.

HDL, high-density lipoprotein; T1WI, T1 weight imaging; HR, hazard ratio; CI, confidence interval.

**Figure 3:**
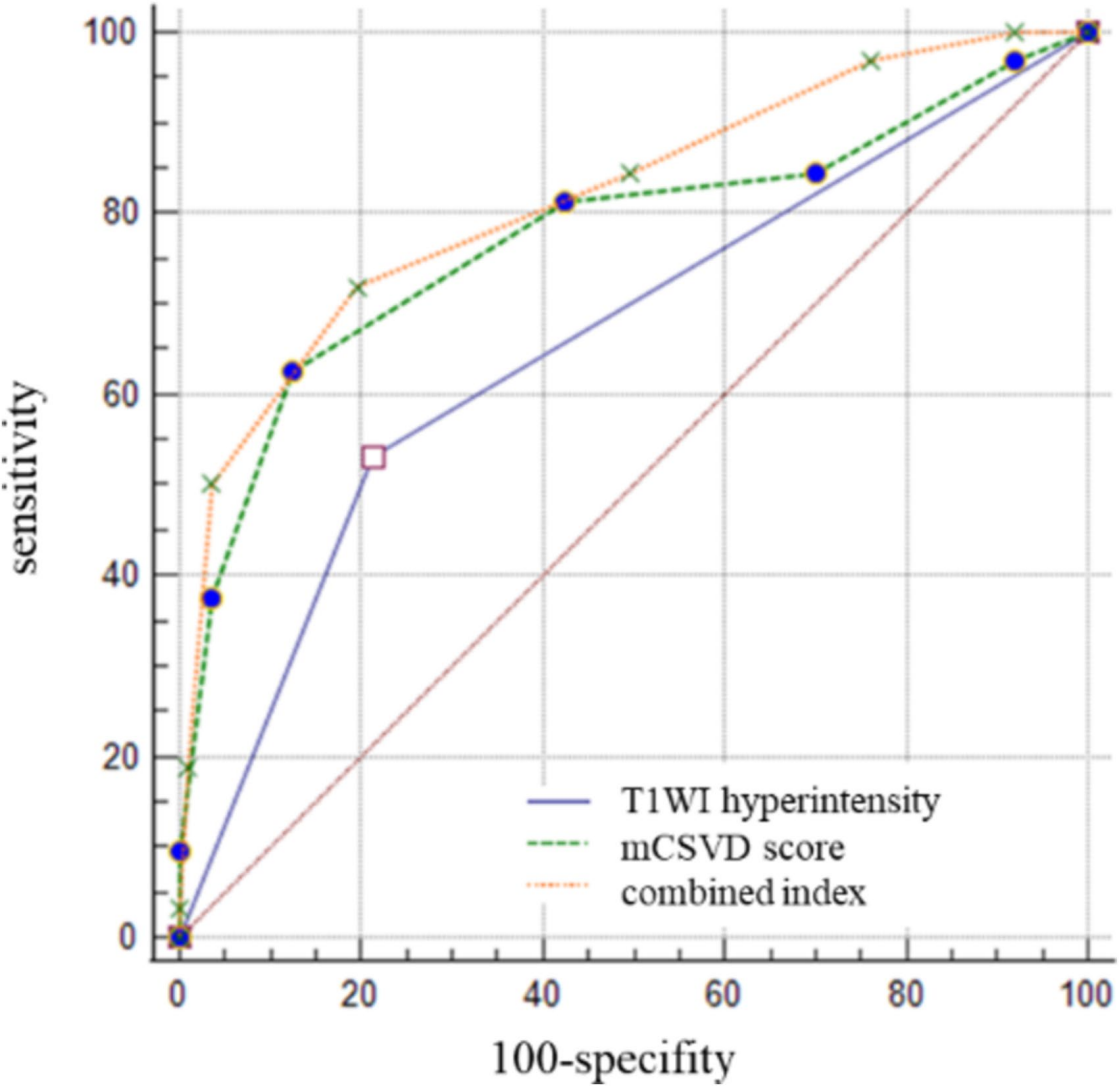
The ROC curve analysis of mCSVD, T1WI hyperintensity, and the combined index in predicting stroke recurrence.

**Figure 4:**
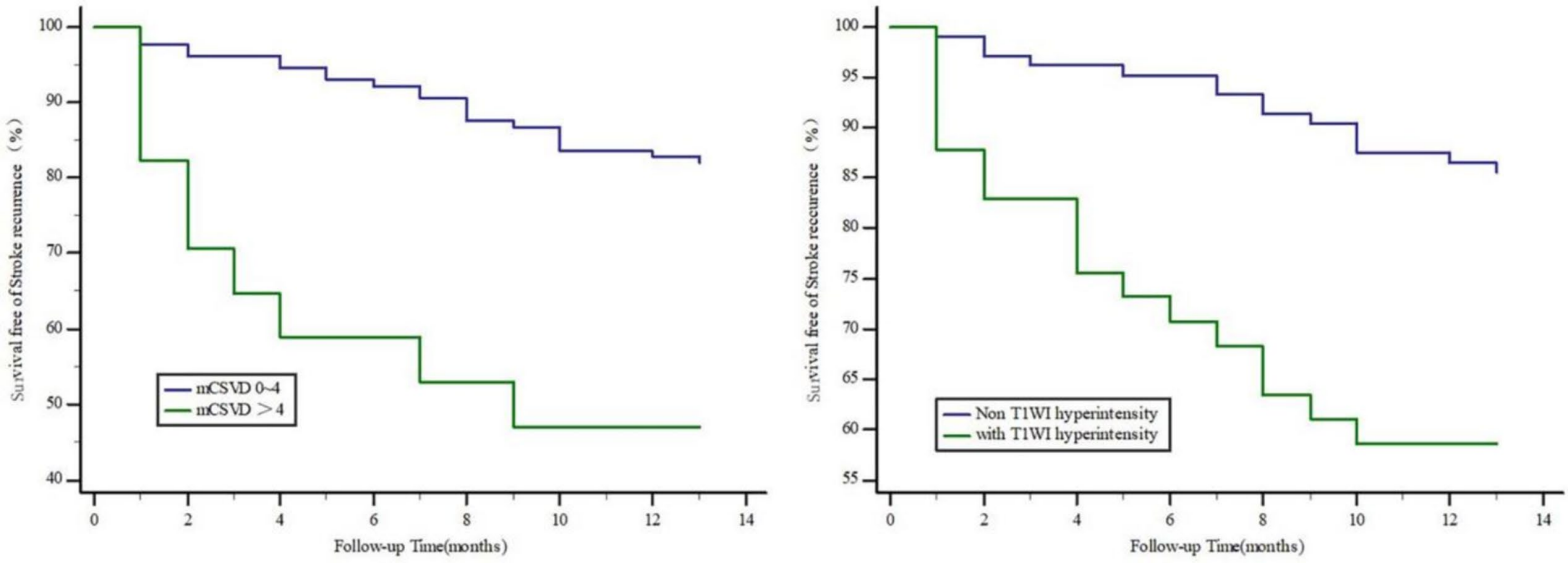
Kaplan–Meier survival curve in patients with stroke recurrence. Patients with mCSVD score > 4 points (*p* < 0.001) and T1WI hyperintensity of the culprit plaque (*p* < 0.001) had a higher risk of stroke recurrence at follow-up.

The cutoff value for the mCSVD score in predicting stroke recurrence was determined to be 4 points, according to ROC curve analysis, which also revealed the AUC value to be 0.775, the sensitivity was equal to 62.50%, and the specificity was 87.51%. The AUC of T1WI hyperintensity was 0.659 for the prediction of stroke recurrence, with a sensitivity and a specificity of 53.13 and 76.76%, respectively. In predicting stroke recurrence, the AUC for the mCSVD score, combined with the T1WI hyperintensity of the culprit plaque, was 0.819. Moreover, the specificity and the sensitivity were 80.53 and 71.87%, respectively, showing the results of the Delong test analysis, revealing the culprit plaque’s T1WI hyperintensity, in conjunction with the mCSVD score, that had the strongest predictive power for stroke recurrence (*z* = 2.678, *p* < 0.05) (Figure 3). The Kaplan–Meier survival curve indicated that individuals with a mCSVD score exceeding 4 points (*p* < 0.001) and T1WI hyperintensity of the culprit plaque (*p* < 0.001) exhibited an elevated risk of stroke recurrence during follow-up (Figure 4).

## Discussion

Intracranial atherosclerotic stenosis and cerebral small vessel disease are essential causes of recurrent stroke patients. However, both follow different pathophysiological mechanisms, but intracerebral atherosclerosis and CSVD typically coexist and share vascular risk factors. Both have a common effect on the occurrence of stroke (15, 16). This study aims to examine the combined effect of two risk variables on stroke recurrence in patients. The study used HR-VWI to determine the type of the culprit plaques in stroke patients. Moreover, the mCSVD score was used to assess the whole brain CSVD disease burden in stroke patients.

Research indicates that individuals with a mCSVD score > 4 points had a 1.730-fold greater chance of experiencing recurrent stroke. The mCSVD score, together with the total CSVD burden score and the severity of prevalent imaging biomarkers of CSVD, offers a reliable assessment of small vessel disease severity across the brain. Initial data from this study indicate the potential effectiveness in predicting stroke recurrence. However, more prospective clinical investigations are necessary to validate these findings. Moreover, stroke patients exhibiting T1WI hyperintensity of the culprit plaque had a 6.568-fold increased risk of recurrence compared to those lacking T1WI hyperintensity. Previous studies on carotid plaques (17, 18) have shown that T1WI hyperintensity of the culprit plaque often signifies the presence of a lipid-rich necrotic core (LRNC) and intraplaque hemorrhage (IPH), with IPH regarded as a component of LRNC. Organizational research studies have demonstrated that plaque fragility is affected by its internal constituents, with LRNCs and IPH potentially increasing the likelihood of plaque rupture and arterial embolism, thereby leading to stroke recurrence (19, 20).

This work has established an integrated prediction model defined by the mCSVD score and T1WI hyperintensity of the culprit plaque. The results reveal that the integrated predictive model has significant effectiveness in predicting stroke recurrence within 1 year among stroke patients. With a specificity of 80.53% and a sensitivity of 71.87%, the combined AUC value of the mCSVD score and the T1WI hyperintensity of culprit plaque was 0.819, greater than any single risk factor, according to the data. Thus demonstrating a robust predictive value. The combined index not only integrated the severity of common imaging subtypes of CSVD but also included the nature of intracranial arterial culprit plaque, which was an important factor influencing the recurrence of stroke. It has a strong clinical and practical value and is simple to promote in clinical environments. This tool can assist clinicians in the preliminary evaluation of recurrence risk in ischemic stroke patients and improve strategies for secondary stroke prevention.

The recurrence group in this study had a higher median mCSVD score, WMH Fazekas grade, CMB number, and number of lacune than the non-recurrence group. According to earlier research, the percentage of WMH detected on MRI in stroke patients might range from 67 to 98% (21). After following up on 7,101 ischemic stroke patients for a year, Ryu et al. verified the correlation between WMH load and hemorrhagic transformation, stroke recurrence, and all-cause mortality. Moreover, the prevalence of WMH in all patients was reported to be 86 and 97% in the group of patients who experienced recurrences (22). However, previous studies have found that the number of cerebral microbleeds is related to cognitive impairment and cerebral hemorrhage after intravenous thrombolysis (23, 24). Large cohort studies are required to investigate the association between CMBs and stroke recurrence in depth. Plaque enhancement, characteristic of susceptible plaques, is associated with capillary growth, inflammation-induced vascular wall stimulation, and endothelial injury (25). Research by Song et al. (4) demonstrates that plaque enhancement independently corresponds with the recurrence of ischemic stroke, with higher enhancement levels increasing the probability of recurrence. In this study, univariate analysis revealed that the proportion of significantly larger plaques in the recurrent group was significantly greater than in the non-recurrent group. In multivariate analysis, the association between stroke recurrence and plaque enhancement was observed to diminish. It was speculated that the differences might be due to Song et al. (4) grading the degree of plaque enhancement. In contrast, plaque enhancement was only a categorical variable in the present study. Therefore, more research is necessary to establish the relationship between plaque enhancement and recurrence.

The study found that the occurrence of diabetes was more common in the recurrence group in comparison to the group without recurrence. Additionally, the recurrence group showed elevated fasting blood glucose levels and glycosylated hemoglobin relative to the group without recurrence. Jiao et al. (26) observed a definitive correlation between blood glucose management and the attributes of cerebral atherosclerotic plaques in individuals diagnosed with type 2 diabetes. Inadequate regulation of blood glucose levels will impact the severity and susceptibility of intracranial atherosclerotic plaques, thereby contributing to the higher risk of stroke recurrence. Further investigation is required to understand the precise mechanism by which controlling blood glucose levels affects the likelihood of stroke recurrence in individuals with type 2 diabetes. It was also observed that, in the recurrent group, the blood HDL level was lowered (*p* < 0.05) in contrast to the non-recurrent group. This difference may be attributed to the anti-atherosclerosis impact of HDL (27).

This study has several limitations. Firstly, regarding the selection of research participants and sample size, patients with severe stroke symptoms were often omitted owing to inadequate image quality, possibly leading to a bias toward patients with less severe stroke presentations. Moreover, the sample size and the follow-up period were limited. Further research will mitigate these limitations by augmenting the sample size and prolonging the follow-up duration to enable a more thorough investigation of stroke recurrence. Secondly, regarding imaging data collection, the 3 T MRI demonstrates limited resolution relative to the 5 T and 7 T MRI systems, making it less effective at illustrating intricate characteristics of plaque components, including plaque surface ulcers and fibrous caps. This study obtained data on plaque thickness, stenosis degree, and burden by manual delineation and semi-automated measuring methods. Despite the execution of repeatability examinations, the possibility of error in the results persists. Future initiatives will focus on augmenting image resolution and reducing measurement errors through integrating higher field strength magnetic resonance machines and deploying artificial intelligence software for the automated segmentation and quantification of diverse plaque components. Thirdly, the efficacy of the prediction model has yet to be confirmed. Future initiatives will entail executing multicenter studies to externally evaluate the study results, aiming to improve clinical practitioners’ ability to detect patients at elevated risk for stroke recurrence in their standard practice.

## Conclusion

Both the mCSVD score and the T1WI hyperintensity of the culprit plaque associated with intracranial arterial were the independent factors related to the risk for stroke recurrence. In conjunction with the T1WI hyperintensity of the culprit plaque, the mCSVD score provides a strong predictive potential for stroke recurrence, allowing for the establishment of a tailored treatment plan and the hierarchical management of sICAS patients.

## Data Availability

The raw data supporting the conclusions of this article will be made available by the authors, without undue reservation.
